# Recent Advances in the Development and Application of Cell-Loaded Collagen Scaffolds

**DOI:** 10.3390/ijms26094009

**Published:** 2025-04-24

**Authors:** Qiming He, Tao Feng, Yingyan Xie, Sathiskumar Swamiappan, Yue Zhou, Yanfang Zhou, Hui Zhou, Xinsheng Peng

**Affiliations:** 1 Dongguan Key Laboratory of Drug Design and Formulation Technology, Biomedical Innovation Center, Guangdong Provincial Key Laboratory of Research and Development of Natural Drugs, and School of Pharmacy, Guangdong Medical University, Dongguan 523808, China; qiminghe30@163.com (Q.H.); christithen@outlook.com (T.F.); xyy765103712@163.com (Y.X.); sathiskumarswamiappan@gmail.com (S.S.); zhouyue12152023@163.com (Y.Z.); 2 Department of Pathophysiology, Guangdong Medical University, Dongguan 523808, China; yfzhou@gdmu.edu.cn

**Keywords:** collagen, cell scaffolds, hydrogel, sponge, tissue engineering, tissue repair

## Abstract

Tissue engineering techniques aim to improve or replace biological tissues or organs by utilizing the extracellular matrix to facilitate the repair of damaged tissues or organs. Collagen-based scaffolds offer numerous advantages, including excellent biocompatibility, low immunogenicity, biodegradability, hemostatic properties, and mechanical strength. Collagen scaffolds can reconstruct the extracellular microenvironment, promote cell adhesion, migration, proliferation, and differentiation, and play a critical role in cell-to-cell and cell-to-matrix interactions. Collagen has been extensively utilized in tissue engineering to facilitate tissue repair and organ reconstruction. This review examines the properties of collagen, including its composition, structure, biological characteristics, and role in regulating various cellular behaviors. Additionally, the preparation of cell-loaded collagen scaffolds is discussed, along with a comprehensive overview of their applications in various tissues, including skin, nerve, bone/cartilage, heart, liver, and others. Emerging strategies and future perspectives for clinical tissue repair are also presented. This review provides a comprehensive synthesis of the mechanisms underlying the use of cell-loaded collagen scaffolds as advanced biomaterials, emphasizing their potential to expand the clinical applications of collagen.

## 1. Introduction

Tissue and organ regeneration after injury or disease remains a significant clinical challenge, as regular healing is a dynamic, complex, and multi-stage process involving various cellular processes and cell-matrix interactions [1]. Although human tissues possess regenerative capabilities, their capacity is limited, particularly in pathological conditions where repair through intrinsic healing mechanisms becomes challenging [2]. In recent years, advances in tissue engineering technology have offered potential solutions to organ shortages, tissue defects, and dysfunctions by constructing scaffolds that mimic the extracellular matrix, delivering oxygen and nutrients to tissues, and providing appropriate biological and physicochemical properties, biocompatibility, and mechanical strength to repair or restore damaged bones, organs, or tissues [3]. An ideal tissue-engineered scaffold comprises three key elements to support cell-tissue regeneration: an appropriate cell source, growth factor, and scaffold biomaterial [4].

Currently, materials commonly used for tissue engineering are categorized into natural polymers (e.g., collagen, gelatin, chitosan, alginate, elastin, and fibronectin, among others) and synthetic polymers (e.g., polyethylene glycol, polyvinyl alcohol, and polysorbate lactone) [5,6]. Collagen, derived from the natural extracellular matrix, exhibits excellent biocompatibility, degradability, and low immunogenicity. It minimizes adverse reactions such as infections, inflammation, and local tissue responses while contributing to cell adhesion, migration, proliferation, and differentiation, thereby promoting tissue repair [7,8,9]. Gelatin, a derivative of collagen, retains the specific biological properties of collagen, exhibiting improved water solubility and processability compared to collagen, and is extensively used in the fabrication of tissue engineering scaffolds. Although it preserves the arginine-glycine-aspartic acid (RGD) sequence, it lacks the active triple-helix structure, thereby influencing certain aspects of cellular behavior regulation [10]. Additionally, collagen can activate coagulation factors to produce thrombin in response to tissue injury, cleave fibrinogen into fibrin, and aggregate platelets to form a thrombus, effectively preventing bleeding. These hemostatic properties have led to its widespread clinical use [11]. Collagen can also be a carrier for delivering drugs or growth factors, enabling stable and sustained release to enhance tissue repair further [12]. Consequently, collagen shows significant potential as a cellular scaffold in regenerative medicine across various tissue engineering applications.

This review provides a brief introduction to collagen, focusing on its structure, biological properties, and effects on the regulation of cellular behavior, as well as outlining the methods of collagen scaffold preparation, detailing its specific applications in tissues such as skin, nerves, bone, heart, and liver, and concluding with an outlook on future research directions in the field of collagen.

## 2. Collagen Sources, Structure and Properties

To date, 40 vertebrate collagen genes have been identified, forming 29 different homo- or heterotrimers. For example, most type I collagens consist of a heterotrimer composed of two α1 chains and one α2 chain, whereas type II, III, and VII collagens exist exclusively as homotrimers [13]. Type I collagens are primarily derived from mammalian skin and tendon tissues, including porcine, bovine, and ovine sources, while type II collagen is predominantly obtained from cartilage tissues of bovine, porcine, and chicken origin [13]. Notably, collagen can also be sourced from marine organisms [14], such as jellyfish, sea urchins, starfish, fish, and sustainable fishery by-products, including fish phosphorus [15,16], fish skin [16], and fish bones. In the human body, collagen constitutes approximately 30% of the total protein content and is distributed across various tissues, including the skin, intervertebral discs [17], tendons [18], bones [19,20], cornea [21] and placenta [22].

**Figure 1:**
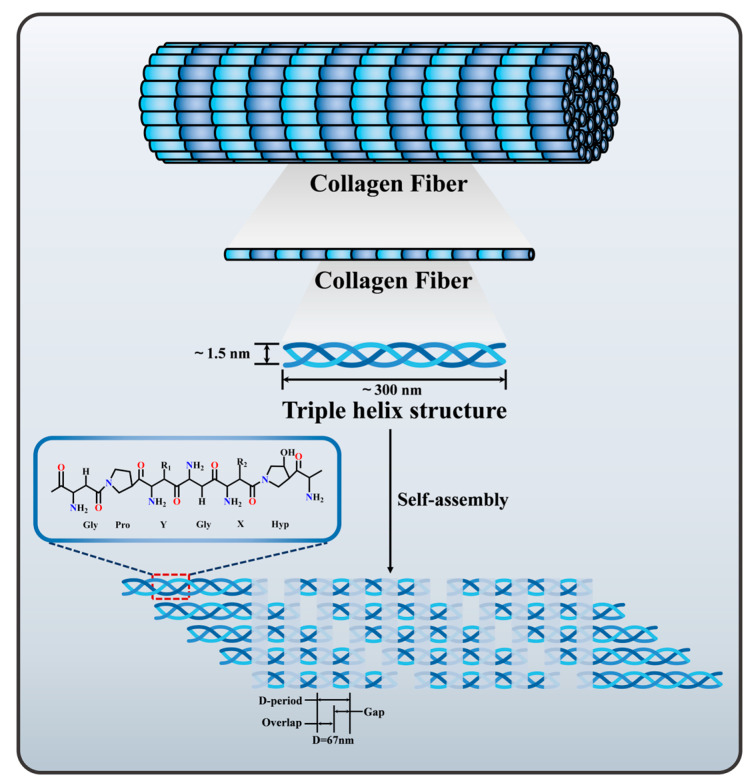
Schematic diagram of collagen fibrillogenesis in vivo.

The primary structure of collagen predominantly consists of the repeating amino acid sequence [Gly-X-Y]n, where proline and hydroxyproline occupy the X and Y positions, respectively; the secondary structure results from the spatial exclusion between proline and hydroxyproline residues at the X and Y positions, forming an α-helix [23]. The tertiary structure, known as tropocollagen, is approximately 300 nm in length, 1.5 nm in diameter, and has a molecular weight of approximately 300 kDa. It is synthesized into a superhelix from three left-handed helical polypeptide chains [24]. Additionally, the tertiary structure forms a collagen microfiber synthesized from tropocollagen, with a staggered quarter-cycle arrangement of the head and tail. These fibers are cross-linked in parallel by covalent bonds and exhibit a band gap in a D-type cycle (approximately 67 nm) [25] (Figure 1).

The primary mechanical strength of collagen arises from its triple helix structure and protofibrils. Structurally, the triple helix adopts an all-trans amide-bonded polyproline type II helix, stabilized by intrachain n→π* interactions between adjacent amide groups and interchain hydrogen bonding between the Gly N-H and Xaa C=O groups [26]. Variations in the Gly-X-Y sequence along the collagen chain length also result in localized differences in helix spacing, kinetics, and thermal stability, thereby affecting the mechanical properties of collagen [27]. Within collagen chains, atoms of individual chains are linked by covalent bonds, while hydrogen bonds, dipole-dipole interactions, ionic bonds, and van der Waals forces connect protofibrils to form fiber bundles [28]. The length, diameter, spatial distribution, type, and molecular weight of collagen fibers collectively determine the mechanical properties of tissues.

In living organisms, matrix metalloproteinases (MMPs) are the only enzymes capable of degrading collagen [29]. Furthermore, the overexpression of MMP-9 in chronic wounds results in delayed wound healing by disrupting the structure of basement membrane proteins, thereby impeding keratinocyte migration, attachment, and epidermal reconstruction [30,31]. Thus, collagen can not only be thoroughly degraded and absorbed by the body but also bind to overexpressed MMPs in wounds to inhibit their activity and prevent the degradation of the extracellular matrix. Additionally, promoting inhibition of tissue inhibitor of metalloproteinase (TIMP) expression and the subsequent inhibition of MMP activity further reduces the inflammatory response [32].

## 3. Effects of Collagen on Cellular Behavior

Collagens interact with cells through various receptors, and their role in regulating cell growth, differentiation, and migration via receptor binding is well-documented. Cell adhesion is a fundamental cellular process that involves the attachment of cells to other cells or inanimate surfaces. Collagen-cellular recognition often occurs through the binding of integrins and discoidin domain receptors (DDRs). The triple-helical collagen ligand GxOGEx’ binds to integrins α1β1, α2β1, α10β1, and α11β1, supporting cellular activity. The strength of cellular adhesion largely depends on the intrinsic affinity between the collagen ligand and the receptor [33,34]. DDR1 and DDR2 are dimeric discoidin domain-containing protein receptors with tyrosine kinase activity, which are activated upon binding to collagen. DDR1 primarily interacts with collagen types I, II, III, and IV, recognizing the collagen triple-helical structure through its discoidin domains. It is widely expressed in epithelial cells, neurons, and macrophages [35,36]. DDR2, in contrast, binds mainly to collagen types I and III and exhibits a high affinity for the GVMGFO motif in collagen [37]. It is predominantly expressed in MSCs, fibroblasts, and chondrocytes [37]. Overexpression of DDR or activation of DDR receptors at the GVMGFO site promotes enhanced integrin-mediated cell adhesion [38]. On the other hand, DDRs mediate cell migration by regulating the cytoskeleton and remodeling the extracellular matrix and protease activity. For example, DDR1 binding to collagen activates the MAPK/ERK pathway, which regulates immune cell migration, monocyte-to-macrophage differentiation, and the secretion of macrophage inflammatory protein-1α and monocyte chemotactic protein-1, further enhancing macrophage migration and chemotaxis [35]. In contrast, smooth muscle cells lacking DDR1 exhibit reduced proliferation, decreased migration, and diminished MMP activity [39]. DDR2 activation primarily regulates MMP expression to mediate cell migration by inducing MMP-8 expression, degrading collagen networks, and releasing collagen-derived chemotactic peptides, thereby inducing neutrophil chemotactic migration in 3D matrices [40]. Human lung fibroblast migration is facilitated through the degradation of the basement membrane by MMP-2 and MMP-10 [41].

In addition, cell migration relies on sensing environmental cues and responding by migrating toward or away from them through mechanisms such as chemotaxis, haptotaxis, durotaxis, and contact guidance [42]. Haptotaxis is triggered by anisotropic alterations in the collagen matrix in vivo, potentially caused by cell-mediated changes in matrix tension or variations in local collagen synthesis and deposition [43,44]. Collagen matrix stiffness influences durotactic migration, where cell clusters migrate along stiffness gradients within the collagen matrix [45]. Cells perceive the topology of collagen fibers within the matrix, resulting in contact-guided migration behavior that enhances the speed and persistence of cell migration [42,46].

Cell proliferation in organisms is influenced by various factors within the extracellular environment and microenvironment, including the vasculature and extracellular matrix. Among these, tissue injury represents a physiological process requiring the proliferation of parenchymal cells to repair damaged tissues. Collagen is the most abundant ECM protein in the dermis and plays a key role in normal tissue homeostasis and skin wound healing [47]. During wound healing, matrix synthesis and remodeling primarily result from the production of collagen and MMPs, particularly MMP-2, by fibroblasts [48]. DDR2 primarily mediates the regulation of collagen, MMP expression, and cell proliferation. Increased DDR2 expression enhances endogenous collagen synthesis, and more collagen can activate DDR2, thereby promoting fibroblast proliferation [49]. It also induces MMP-2 expression, which facilitates the degradation of the damaged matrix, leading to the release of growth factors and chemokines that further stimulate fibroblast proliferation and ECM remodeling [37,49,50]. Studies have shown that DDR2-deficient mice exhibit delayed wound closure, reduced fibroblast recruitment, decreased tensile strength, and diminished collagen content in skin wound healing [51]. Moreover, dysregulated DDR1 expression is associated with impaired wound healing, as keloid fibroblasts express higher levels of DDR1 than normal fibroblasts, potentially contributing to keloid formation by promoting excessive collagen deposition [48].

Specifically, the application of collagen dressing releases degradation products that promote fibroblast proliferation [52]. Additionally, activated platelets or inflammatory cells release MMP-1, which cleaves collagen telopeptides to expose bioactive sites on collagen fibers, enabling immune cells and fibroblasts to migrate and proliferate toward the injured tissues in response to chemokines and cytokines [53]. Meanwhile, macrophages provide a sustained source of growth factors that stimulate fibroplasia and angiogenesis, thereby promoting endothelial cell proliferation for the repair of damaged tissues [54].

Stem cells are undifferentiated, immature, self-replicating cells, including pluripotent stem cells, embryonic stem cells (ESCs), mesenchymal stem cells (MSCs), hematopoietic stem cells, and neural stem cells (NSCs), whose differentiation is influenced by multiple factors [55]. These factors include structural properties such as matrix stiffness, pore structure, and scaffold fiber diameter. For example, collagen hydrogels can stimulate the upregulation of SOX-9 expression, thereby mediating the differentiation of early-cohesion-stage MSCs into chondrocytes via ROCK-dependent actinomyosin contraction [56]. Additionally, collagen influences the differentiation behavior of other cell types, such as human dermal fibroblasts (NHDFs) [57] and mouse adult myoblasts (C2C12) [58].

## 4. Collagen Scaffold Preparation

### 4.1. Chemical Cross-Linking

Chemically cross-linked hydrogels are formed through covalent bonding between polymer chains via various reactions, including Schiff base formation, Michael addition, condensation, ‘click’ chemistry, UV cross-linking, free-radical polymerization, and enzyme-induced cross-linking [59]. They have enhanced mechanical properties and stability compared to physically cross-linked hydrogels.

#### 4.1.1. Click Chemistry

‘Click chemistry’ is a combinatorial chemistry strategy focusing on synthesizing carbon-heteroatom (C-X-C) bonds. Key reaction types in this approach include cycloaddition, nucleophilic ring-opening, the carbonyl chemistry of non-alcoholic aldehydes, and the addition of carbon-carbon double or triple bonds [60].

Thiol-alkene click chemistry activates thiols (-SH) through light or thermal initiators, leading to the formation of sulfur radicals. This process facilitates the Michael addition of olefinic double bonds (C=C) via free radical chain reactions. Alternatively, under alkaline conditions, the thiolate anion (R-S^⁻^) undergoes a nucleophilic attack on the activated olefinic double bond, resulting in the formation of a thioether bond. This reaction exhibits high efficiency but necessitates the incorporation of click-functional groups into natural macromolecules [60,61]. Pupkaite et al. [62] utilized thiol-modified collagen and PEG-maleimide through a Michael addition reaction to form an injectable hydrogel. The hydrogel-encapsulated bone marrow-derived stem cells (BMSCs) and human umbilical vein endothelial cells (HUVECs) maintained over 90% survival. Xu et al. [61] developed hydrogels composed of thiol-modified collagen and acrylate copolymers through a Michael addition reaction, which enabled the encapsulation of BMSCs, facilitating rapid diffusion and the formation of cellular networks.

The copper-free click chemistry reaction, strain-promoted azide-alkyne cycloaddition (SPAAC), proceeds through the nucleophilic attack of azide (N_3_^−^) on the carbon atoms of an alkyne, forming a five-membered ring via a [3 + 2] cycloaddition reaction. This reaction is devoid of copper toxicity, enabling scaffold fabrication in the presence of living cells and facilitating in-situ cross-linking of living tissues [60,63].Chen et al. [64] Cross-linked collagen via a SPAAC reaction and hyaluronic acid via a Michael addition reaction and combined the two to form an interpenetrating polymer network hydrogel. This hydrogel was inoculated with corneal epithelial cells, achieving a 99% survival rate. Epidermal growth factor (EGF) can be conjugated and immobilized onto the collagen surface via the SPAAC reaction, enhancing the adhesion and proliferation of corneal epithelial cells [65]. This reaction may also facilitate the introduction of specific functional groups into collagen, enabling efficient gelation.

#### 4.1.2. Photoactivated Cross-Linking

Photoactivated cross-linking offers the advantage of rapidly forming hydrogel networks under mild conditions. Moreover, the physicochemical properties of the hydrogel, such as pore size and elastic modulus, can be tailored by adjusting the duration and intensity of light exposure, as well as the concentration of the photoinitiator [66].

Methacrylate groups containing conjugated double bonds are commonly introduced into natural macromolecules to provide photosensitive cross-linking sites. Upon UV exposure, photoinitiators cleave to generate free radicals, which subsequently attack unsaturated conjugated double bonds, triggering chain polymerization reactions that lead to the formation of covalent cross-linked networks [66]. He et al. [67] synthesized light-cured collagen methacrylate (COLMA) and chondroitin methacrylate (CSMA) by modifying type I collagen and chondroitin sulfate with methacrylic anhydride. This process resulted in a photo-cross-linked two-component CSMA-COLMA hydrogel matrix with an average storage modulus of 3.3 kPa and an elastic modulus of 30.3 kPa. Keratinocytes and fibroblasts encapsulated in the hydrogel expressed characteristic proteins. In addition, this system can also cross-link type II collagen (Col-II-MA), thereby promoting cellular morphological changes, BMSC proliferation, cartilage formation, and upregulation of chondrogenic gene expression [68].

Riboflavin (RF), also known as vitamin B2, is a cross-linking agent and photoinitiator for UV curing. RF transitions into an excited singlet state upon absorption of UV light energy, subsequently undergoing an intersystemic crossover mechanism to form the excited triplet state. When RF absorbs energy from ultraviolet light, it changes into an excited singlet state, undergoing intersystem crossing to form an excited triplet state and directly interacting with oxygen, generating singlet oxygen radicals. These radicals further oxidize collagen, forming intramolecular and intermolecular covalent cross-links between collagen amino acids [69,70]. Compared to methacrylic anhydride, this approach eliminates the need for photoinitiators, making it milder and more biologically safe; however, its mechanical properties are limited. Fan et al. [70] compared the bioactivity of fibroblasts in collagen matrices cross-linked by EDC/NHS, kyonipin, and RF. They found that RF-cross-linked matrices, which did not involve chemical cross-linking agents, exhibited a cell survival rate exceeding 80%, whereas chemically cross-linked collagen matrices exhibited some cytotoxic effects. In another study, BMSCs were encapsulated in a collagen/hyaluronic acid liquid mixture containing RF, which rapidly polymerized into a gel under blue light irradiation within three minutes [71].

#### 4.1.3. Enzyme Cross-Linking

Enzyme cross-linking offers several advantages, including mild reaction conditions, minimal by-product formation, high specificity, and high yield. The key enzymes utilized in this process include lysine oxidase (LOX), glutamine transaminase (MTG), and horseradish peroxidase (HRP), which primarily target amino groups. Under physiological conditions, the post-translational modification of these enzymes on collagen helps maintain its stability, elasticity, and bioactivity. The strength of the gel can be regulated by adjusting the enzyme concentration, facilitating the formation of robust covalent bonds and promoting rapid gelation [72,73].

LOX is an enzyme primarily reliant on copper ions to catalyze the oxidative deamination of the ε-amino groups of lysine or hydroxylysine residues in collagen, generating aldehyde groups that condense to form stable aldimine cross-links [74,75]. This process primarily occurs during in vivo biosynthesis and is limited by the reaction rate, making it less commonly applied in the synthesis of biomaterials.

HRP utilizes H_2_O_2_ as an oxidant, catalyzing the formation of free radical couplings through tyrosine, histidine, or lysine residues, which enables rapid and non-specific cross-linking [76]. Ying et al. [73] cross-linked collagen and tyrosinated hyaluronic acid using HRP and H_2_O_2_ to form a hydrogel, in which encapsulated human microvascular endothelial cells (HMECs) and fibroblasts exhibited significant proliferative activity.

MTG possesses high catalytic efficiency and is widely employed to enhance the mechanical strength of collagen, which catalyzes the acyl transfer reaction between the γ-carboxamido group of glutamine residues (the acyl donor) and the ε-amino group of lysine residues (the acyl acceptor) in collagen peptide chains, leading to the formation of both inter- and intramolecular ε-(γ-glutamyl)-lysine covalent bonds [77]. For example, Tayabally et al. [78] cross-linked collagen with transglutaminase to form hydrogels, which provided an ideal environment for the three-dimensional culture of human dental pulp stem cells (HDPSCs). While an increased concentration of MTG promoted the formation of collagen fibers, it simultaneously enhanced the adhesion and proliferation of fibroblasts in the hydrogel [77].

### 4.2. Physical Force

Physically cross-linked hydrogels are primarily formed through reversible molecular interactions, including electrostatic interactions, hydrogen bonding, hydrophobic/hydrophilic interactions, crystallization, self-assembly, and thermally induced chain entanglement. A significant advantage of these hydrogels is their biosafety, as they do not necessitate the introduction of chemical cross-linking agents, thereby mitigating potential cytotoxicity and other related concerns [79].

**Figure 2:**
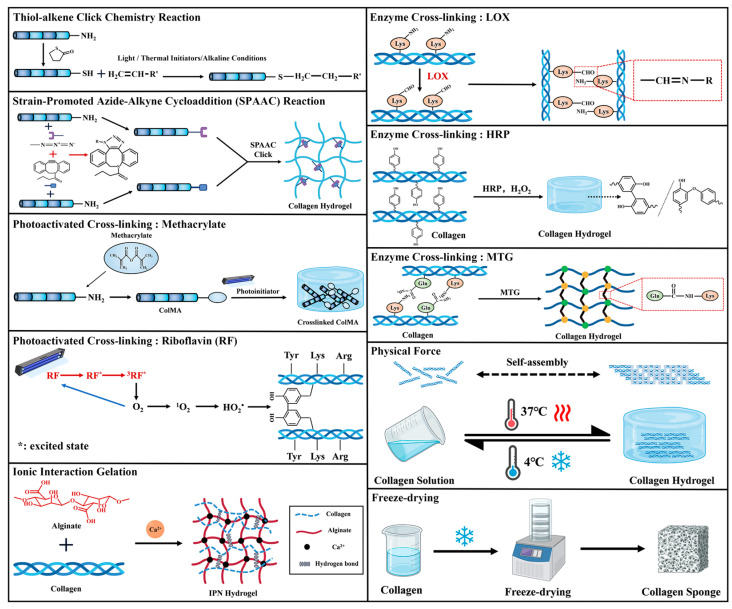
Schematic diagram of the preparation methods of cell-loaded collagen scaffolds.

Temperature-induced phase transition hydrogels are modulated by varying the ratios of hydrophilic and hydrophobic components, resulting in differential solubilities at specific temperatures. These transitions are characterized by the Low Critical Solution Temperature (LCST) and Upper Critical Solution Temperature (UCST) [80]. Du et al. [81] employed simulated body fluid (SBF) to prepare a pH-neutral, rapid-sol-gel reversible thermosensitive collagen (RRTC) hydrogel. This material gelled into a hydrogel within 60 s at 37 °C and reverted to a collagen solution at 4 °C. The hydrogel effectively encapsulated various cells, including mouse fibroblasts (L929), HUVECs, mouse myeloma cells (Sp2/0), murine aortic vascular smooth muscle cells (MOVAS), rat intestinal epithelial cells (IEC-6), and human chondrocytes (HCs), resulting in approximately a ten-fold increase in proliferation over 14 days. Huang et al. [56] pre-polymerized neutral collagen, incubated at 4 °C and then at 37 °C for 30 min to form a hydrogel. The self-assembly of the collagen fiber network was controlled by adjusting the pre-polymerization time at 4 °C. As the incubation time at low temperatures increased, the diameter of the collagen fibers and their self-assembly progressively improved, leading to a more organized and interconnected collagen fiber network. Additionally, collagen fiber alignment was optimized using a low-temperature ammonia vapor technique. Collagen solution was introduced into a homemade freeze-casting device, where collagen fiber formation was induced at 0 °C. After 48 h, the collagen was immersed in 5× PBS to cause swelling, resulting in a fibrous collagen scaffold with high porosity, anisotropic properties, and enhanced cellular adhesion. This scaffold was then inoculated with NHDFs and C2C12 [82]. Unfortunately, the mechanical properties of self-assembled gels formed through temperature-induced phase transitions are insufficient to meet the mechanical demands of hard tissues (Figure 2).

### 4.3. Ionic Interaction Gelation

Sodium alginate is a naturally occurring polysaccharide-based polymer known for its unique gelation properties in aqueous solutions when exposed to divalent cations (e.g., Ca^2^⁺, Ba^2^⁺). These properties closely resemble those of a natural extracellular matrix, allowing for the encapsulation of cells in a highly hydrated, three-dimensional environment [83]. However, sodium alginate hydrogels possess certain limitations, including instability and fragility. Specifically, (1) the mechanical properties are insufficient to provide adequate support in stress-induced environments, and (2) the gel lacks adequate cell adhesion sites, resulting in suboptimal cellular interactions. Interpenetrating Polymer Network (IPN) technology can address these limitations. An IPN hydrogel is a three-dimensional network structure formed by two or more interpenetrating and entangled, cross-linked polymers. This structure has been extensively utilized as a scaffolding material in various biomedical applications, including tissue engineering and drug delivery systems [84]. Collagen, renowned for its exceptional cell adhesion properties, facilitates the migration of individual cells or cell clusters. Additionally, its abundance of functional groups (e.g., hydroxyl, carboxyl, and amino groups) promotes the formation of hydrogen bonds with sodium alginate, creating an interpenetrating network. This interaction enhances the three-dimensional mesh structure of the co-blended hydrogel, improving its mechanical properties, promoting cell adhesion, and facilitating cell proliferation and migration [85].

Calcium ions (Ca^2^⁺) play a pivotal role in modulating the stiffness of hydrogels. Bernero et al. [86] cross-linked fast-relaxing and slow-relaxing alginate-collagen IPN hydrogels using 20 mM CaCl_2_ and 5 mM CaCl_2_, respectively. Their study revealed that the fast-relaxing gel promoted the early proliferation of murine osteoblasts (IDG-SW3), whereas the slow-relaxing gel facilitated osteogenic differentiation. Due to its ability to adjust stiffness, sodium alginate/collagen gel precursor solutions were also employed in microgel printing, incorporating the human fibroblast cell line (HS-5) and the epithelial adenocarcinoma cell line (MDA-MB-231). Adding Ca^2^⁺ subsequently induced gelation, enabling stiffness-adjustable 3D cell printing [87].

Alginate/collagen IPN network hydrogels provide a robust scaffold for facilitating diverse forms of cell growth, as demonstrated by Zhang et al. [88]. Human umbilical cord mesenchymal stem cells (hUC-MSCs) were employed to treat skin wounds by loading them onto these hydrogels. Furthermore, human osteosarcoma cells (MG-63), human osteosarcoma clonal cells (U2OS), epithelial adenocarcinoma cells (MDA-MB-231), and human colon cancer cells (HCT-116) were encapsulated within the hydrogels to construct 3D in vivo microenvironments for the study of cell migration [89].

Overall, the combination of collagen and alginate leverages the advantages of both materials, particularly the ability to preserve cellular activity during rapid gelation via ionic interactions. This synergy holds significant potential for developing cell-loaded structures in tissue regeneration engineering.

### 4.4. Freeze-Drying

Porous sponge scaffolds are considered ideal natural scaffolds due to their interconnected structure, which provides mechanical support and promotes cell adhesion and migration. These scaffolds are typically fabricated via a freeze-drying process, which effectively preserves the active triple-helical structure of collagen, as it does not require the addition of exogenous chemical reagents and is maintained at low temperatures. Additionally, pore size, morphology, and distribution can be precisely controlled by adjusting the freezing rate, freeze-drying temperature, and polymer concentration [90,91]. However, sponge scaffolds exhibit inherent limitations due to process constraints: (1) Their mechanical strength is insufficient to withstand significant mechanical loads in hydrated environments, restricting their application in tissues with high mechanical demands. (2) The degradation rate of these scaffolds is relatively rapid in comparison to other cross-linking methods, with a single collagen sponge completely degrading within weeks in an enzymatic in vivo environment. (3) Challenges related to long-term stability and storage arise, as sponge scaffolds are prone to moisture absorption, which can result in structural softening, collapse, and compromised performance in implanted tissue applications. Consequently, careful storage is necessary to maintain dryness and preserve their structural integrity [92,93].

To improve mechanical strength and degradation rate, cross-linkers or other biomaterials are often introduced to enhance the physicochemical properties of scaffolds. Khadivar et al. [94] freeze-dried type I collagen, along with nano-fibrillar cellulose and carboxymethyl diethylaminoethyl cellulose, to obtain porous sponges, resulting in scaffolds with enhanced tensile strength and improved stability. These scaffolds were subsequently loaded with BMSCs and differentiated keratinocytes for the repair of full-thickness wound injuries. Zhao et al. [95] cross-linked collagen with bialdehyde carboxymethyl cellulose to enhance mechanical properties and optimize pore density. The resulting sponges formed a bilayer structure, with a dense upper layer supporting the proliferation of C2C12 myoblasts and a porous lower layer promoting the growth of osteoblasts MC3T3-E1. Table 1 summarizes the various cell types and preparation methods for collagen scaffold encapsulation. Table 2 summarizes the advantages and disadvantages of preparation methods for cell-loaded collagen scaffolds.

## 5. Cell-Loaded Collagen Scaffolds for Tissue Engineering Applications

### 5.1. Skin Tissue Engineering

Skin trauma disrupts the normal anatomical structure and function, often resulting from exposure to external factors such as chemicals or environmental hazards. The physiological process of wound healing is divided into four phases: hemostasis, inflammation, proliferation, and remodeling [52]. Currently, three main strategies in skin tissue engineering have been identified: (1) cell patches or solutions composed entirely of cells; (2) 3D scaffolds fabricated from synthetic or natural polymers; (3) 3D scaffolds and dermal substitutes co-cultured with cells in vitro. Collagen plays a crucial role in wound healing by supporting hemostasis, mitigating inflammation, inducing angiogenesis, and aiding tissue regeneration. Collagen enhances the healing process by maintaining cell viability and improving retention at the wound site when combined with stem cells. For instance, Lashkari et al. [96] developed a bilayer nanofiber scaffold dressing for full-thickness wounds in rats using electrospinning to deposit polycaprolactone/gelatin (PCL/Gel) nanofibers onto a collagen/alginate (Col/Alg) hydrogel. The dressing was subsequently seeded with adipose-derived stem cells (ADSCs), which alleviated inflammation, enhanced epithelialization, and induced collagen regeneration. Similarly, Khadivar et al. [94] used bone marrow-derived MSCs, differentiated into keratinocyte-like cells, in conjunction with skin scaffolds made of type I collagen, carboxymethyl diethylaminoethyl cellulose, and nanofibrillar cellulose to accelerate the healing of full-thickness wounds in rats. The same can be applied to chronic wounds. Through enhanced vascularization and paracrine signaling, 3D MSC spheroids combined with an injectable temperature-sensitive collagen/chitosan/β-glycerophosphate hydrogel accelerated the healing of diabetic wounds [97].

Collagen is also a viable option for dermal replacement matrix material, possessing a 3D structure similar to that of the dermis and the ability to be rapidly vascularized and appropriately degraded, thereby maintaining optimal strength and toughness. Liu et al. [98] implanted BMSCs into collagen dermal replacement scaffolds (CDRSs) to create bioactive skin substitutes for wound healing applications. These scaffolds effectively modulated the polarization of M1-type macrophages, supporting non-contractile epithelialization, granulation tissue formation, and neovascularization in diabetic wounds. These scaffolds are promising for treating chronic wounds and preventing disease recurrence. Xie et al. [99] inoculated MSCs into collagen/glycosaminoglycan matrices to engineer dermal-like tissue sheets (EDSs). When applied to wounds in diabetic and healthy mice, MSCs in the EDSs exhibited improved preservation compared to those in standalone collagen hydrogels or matrices. The EDSs retained a higher density of MSCs, enhanced their activity, and extended their presence at the wound site. Both collagen dermal substitutes induced the expression of chemokines and pro-angiogenic factors in injured tissue, facilitating chronic wound healing.

### 5.2. Neural Tissue Engineering

Injuries to the central nervous system (CNS) are a leading cause of disability worldwide and profoundly impact patients’ quality of life; however, effective therapeutic options for prediction and treatment remain lacking. Recent progress in neural tissue regeneration engineering has facilitated the development of novel therapeutic paradigms for neurodegenerative disorders, including Alzheimer’s disease, traumatic brain injury (TBI), and spinal cord injury (SCI) [100]. The restricted migratory capacity of exogenous cells toward the injury site hinders the in situ proliferation of neural stem cells. Collagen, however, serves as a scaffold for endogenous and transplanted cells, supporting cell growth and providing directional guidance for neural development [101]. In recent years, integrating transplantable biomaterials with cell-based therapies has emerged as a promising therapeutic strategy.

TBI results from external mechanical forces that induce tissue strain or deformation, leading to secondary brain damage. This secondary injury is characterized by inflammation, necrosis, excitotoxicity, oxidative stress, and vascular disruption. As these delayed responses progress, they aggravate neurodegeneration and contribute to increased cell death [102]. Collagen scaffolds mitigate cell inactivation due to insufficient tissue support by enabling the controlled transport of neural stem cells to the injury site and promoting their growth in the affected area. Kim et al. [103] reported that a novel delivery method using collagen/fibronectin hydrogel for delivering mouse neural stem cell (mNSC) spheroids maintained mNSC viability, reduced neuronal degeneration, and enhanced cognitive function. Treated mice exhibited a significant increase in preference for novel objects, demonstrating improved cognitive performance through the reconstruction of the damaged cortex to treat mild TBI. Zhang et al. [104] utilized collagen/heparan sulfate porous scaffolds embedded with neural stem cells (NSCs) to treat a rat model of TBI induced by controlled cortical impingement. Following treatment, marked improvements were observed in the injured brain tissue, including enhanced nerve fibers, synapses, and myelin regeneration. Furthermore, brain edema and apoptosis were substantially reduced, resulting in a significant restoration of motor and cognitive functions in the rats.

The intervertebral disc consists of an inner nucleus pulposus (NP), primarily made up of type I collagen, and an outer annulus fibrosus (AF), which comprises a meshwork of type II collagen, water, and proteoglycans [105]. Strategies to reduce vascularization in the NP tissue have demonstrated potential in mitigating intervertebral disc degeneration (IDD)—for instance, Hu et al. [106] developed an in situ injectable collagen/methacrylate hyaluronic acid hydrogel that encapsulates MSCs and incorporates the vascular endothelial growth factor receptor inhibitor cabozantinib. This hydrogel-maintained disc height protected nerve endings and reduced vascularization and inflammation in a rat model of caudal IDD induced by puncture. AF defects majorly contribute to recurrent disc herniation and post-discectomy degeneration. In an animal model of caudal disc AF defects, administration of an injectable collagen hydrogel containing MSCs significantly enhanced the MRI index, preserved disc structure, minimized apoptosis, and promoted anti-inflammatory expression [107].

SCI typically results in permanent disability as well as motor and sensory deficits. Current treatment strategies primarily focus on reducing spinal cord swelling by preventing cell death, minimizing oxidative stress, blocking secondary damage, and removing damaged bone, disc, and ligament fragments. However, these approaches remain insufficient for promoting axonal regeneration [108]. Collagen scaffolds have emerged as promising tools for facilitating cell transport and bridging devices to re-establish continuity in the injured region, promoting neuronal regeneration. Zou et al. [109] inoculated human fetal brain neural stem cells (hbNSPCs) and spinal cord-derived neural stem cells (hscNSPCs) onto oriented collagen sponge scaffolds, followed by transplantation into rats’ completely transected spinal cords. hscNSPC-ACSS effectively promoted neuronal regeneration, myelin sheath formation around nerve fibers, and synaptogenesis. This scaffold structure substantially improved motor function recovery and optimized the microenvironment at the injury site. Furthermore, a three-dimensional collagen/silk protein scaffold, designed to mimic the anatomical structure of the spinal cord lumen, was found to support cell growth under both in vivo and in vitro conditions. This scaffold enables the targeted delivery of NSCs, enhances spinal cord continuity, and effectively fills the injury cavity [110].

### 5.3. Bone/Cartilage Tissue Engineering

Bone tissue, the hard connective tissue of the human body, provides structural support and protects delicate internal organs. Severe bone defects can result from trauma, infection, congenital malformations, osteogenesis imperfecta, and osteoporosis [111]. While bone tissue is inherently self-repairing and regenerative, minor defects usually heal without medical intervention. However, natural regeneration becomes insufficient when bone defects surpass a critical size. The efficient regeneration, mineralization, and healing of bone tissue depend on its dynamic remodeling processes, which are governed by intricate cellular mechanisms. The most common clinical approaches for treating bone defects are surgical reconstruction and bone grafting [112]. In recent years, advancements in bone tissue engineering have introduced innovative solutions for addressing bone defects, surpassing the limitations of traditional bone healing and reconstruction techniques [113].

Bone tissue scaffolds must meet three essential criteria: osteoinductivity, osteoconductivity, and biocompatibility [114]. Collagen, a vital component of the bone matrix, plays a critical role in bone formation. Existing research has demonstrated that collagen plays a crucial role in the mineralization process, and mineralized collagen facilitates bone regeneration through its osteoconductive properties and inherent bioactivity. Beyond serving as a structural substitute for bone tissue, collagen promotes stem cell proliferation, enhances osteogenic differentiation, and suppresses osteoclast differentiation [115].

Collagen is often combined with ceramic materials, such as hydroxyapatite and bioactive glass, to improve the scaffold’s osteoinductive and osteoconductive properties [116]. Guo et al. [117] utilized 3D printing to incorporate BMSCs and hydroxyapatite (HAP) into collagen scaffolds designed to mimic the composition of bone. This approach enhanced alkaline phosphatase expression and generated bioactive substances that promote bone formation, suggesting potential applications in the treatment of bone defect diseases. Calabrese et al. [118] incorporated human adipose-derived stem cells (hADSCs) into collagen/HAP scaffolds, thereby enhancing osteogenic differentiation, bone formation, and angiogenic potential. In another study, collagen/HAP/strontium-doped mesoporous bioactive glass scaffolds were inoculated with osteoblasts (OBs) derived from bone trabeculae and osteoclast precursors (OCs) from hematopoietic samples. These cells adhered to the scaffold surface through filamentous pseudopodia and membrane protrusions, further enhancing ALP expression [119].

Furthermore, collagen can be combined with synthetic polymers such as polycaprolactone (PCL), poly (vinyl alcohol) (PVA), and polylactic acid-hydroxyacetic acid copolymer (PLGA) to improve mechanical strength and stability [116,120]. Liu et al. [121] seeded BMSCs onto collagen/PVA-CaCO₃ membranes, which effectively promoted the adhesion, proliferation, and osteogenic differentiation of BMSCs. Yang et al. [122] seeded MSCs onto collagen/PLGA/HAP fibrous scaffolds, where the collagen coating enhanced cell-membrane interactions, promoted cell spreading, and increased alkaline phosphatase (ALP) activity along with osteogenic gene expression. Collagen/phosphorus-modified polycaprolactone (P-PCL) bioactive scaffolds inoculated with adipose-derived mesenchymal stem cells (AD-MSCs) exhibited superior ALP activity and increased mineralized nodule formation, enhancing osteoinductivity [123]. Despite these promising results, the studies above did not utilize bone defect models, which restricts their clinical relevance.

Articular cartilage repair is limited in self-renewal capacity because it lacks blood vessels, lymph nodes, and nerves. The structural integrity of the collagen network plays a crucial role in the effectiveness of cartilage regeneration scaffolds. Type I collagen has been shown to create an ideal physiological microenvironment for cartilage formation by BMSCs and to support the reconstruction of both superficial and calcified cartilage. A collagen-decellularized extracellular matrix (dECM) scaffold was fabricated by integrating collagen with dECM derived from immature cartilage tissues at distinct developmental stages. This scaffold promoted the differentiation of BMSCs into fibrochondrocytes [124]. Additionally, Yu et al. [125] employed ADSCs embedded in collagen/silk protein scaffolds in a New Zealand rabbit model of cartilage defect. This approach significantly enhanced cartilage regeneration, facilitating the gradual replacement of cartilage with newly formed cartilage tissue.

### 5.4. Cardiac Tissue Engineering

In humans, the myocardium’s extracellular matrix (ECM) comprises approximately 75% to 80% fibrous collagen, predominantly types I and III, synthesized by cardiac fibroblasts. This collagen provides elasticity and structural integrity to cardiac tissues, interacts with integrins to mediate cell adhesion, and supports the shape, thickness, and stiffness of the myocardium throughout the cardiac deformation cycle [126]. Cardiovascular diseases, particularly myocardial infarction (MI), are the leading cause of death globally. MI results from the acute obstruction of coronary arteries, leading to an inadequate blood supply to the corresponding myocardial region, which causes necrosis and dysfunction of heart tissue. Current therapeutic approaches primarily focus on limiting MI by restoring blood flow to the ischemic myocardium through reperfusion techniques, including coronary intervention, thrombolysis, and coronary artery bypass grafting. However, these interventions are insufficient in stimulating the repair and regeneration of damaged myocardial tissue. Promoting myocardial regeneration in vivo remains a central objective in cardiac tissue engineering [127].

Conductive materials play a critical role in cardiac tissue engineering. However, the sole use of electroactive biomaterials is often incompatible with cardiac tissue growth due to their degradation rates and mechanical properties. Conversely, collagen can serve as a scaffold at defective sites, maintaining structural integrity, providing cellular attachment domains, and replicating the microenvironment necessary for cardiomyocyte growth [128]. Roshanbinfar et al. [129] employed the conductive material PEDOT–PSS (poly(3,4-ethylenedioxythiophene) polystyrene sulfonate) to modify the micromorphology of collagen hydrogel single fibers. The resulting morphology exhibited coarser, tangled fibers interspersed with finer individual fibers, resembling the natural cardiac ECM structure. Human-induced pluripotent stem cell (hiPSC)-derived cardiomyocytes within collagen-PEDOT–PSS hydrogels exhibited near-adult myonode lengths, enhanced contractility, improved calcium handling, and increased conduction velocity. Intracardiac injection into infarcted mice protected the infarcted myocardium from induced ventricular tachycardia. Tohidi et al. [130] incorporated gold nanoparticles and (3-Aminopropyl)triethoxysilane (APTES)-grafted oxidized bacterial cellulose into hydrogels functionalized with hyaluronic acid and collagen. Hydrogels loaded with human embryonic stem cell-derived cardiomyocytes underwent electrical resistance measurements, yielding a value of approximately 0.1 S/m. This electrical conductivity is suitable for electrical stimulation, confirming the potential of these hydrogels as scaffolds for the treatment of MI.

In recent years, 3D printing technology has emerged as a promising strategy for creating highly controlled 3D tissue environments that closely replicate natural tissues. Kim et al. [131] employed collagen/methacryloylated gelatin hydrogel as a bio-ink for encapsulating human ventricular cardiomyocytes (hCMs) and cardiac fibroblasts (hCFs). By comparing two preparation methods—3D bioprinting and manual inoculation via pipette—they observed that the hydrogel used in 3D bioprinting had a more porous structure, which enhanced cell viability. The transplantation of 3D-printed patches into rats with acute myocardial infarction (AMI) enhanced long-term graft survival, vascularization, and stability, reduced fibrosis, increased left ventricular thickness, and improved cardiac function. Additionally, limitations may arise in encapsulating cardiomyocytes due to the mechanical modulus of a single collagen fraction [132]. To overcome this limitation, the Freeform Reversible Embedding of Suspended Hydrogels (FRESH) method was proposed for the 3D bioprinting of collagen. Lee et al. [133] demonstrated that this technique enables precise control over the composition and microstructure of collagen, allowing for the design of human cardiac constructs that replicate the heart’s structure, from capillaries to the entire organ. Using this method, human cardiomyocytes were encapsulated in 3D-printed ventricles, which exhibited synchronized contraction during peak systolic periods, directed action potential propagation, and up to 14% wall thickening.

### 5.5. Liver Tissue Engineering

The liver is a vital organ with many essential functions in the human body and exhibits substantial regenerative capacity. However, severe injury can impair this regenerative capacity. Dysfunctions resulting from factors such as drugs, toxins, viral infections, and cancers can lead to end-stage liver disease or acute liver failure, with in situ liver transplantation remaining the only effective treatment [134]. Liver tissue engineering has emerged as a promising solution to address the shortage of liver transplants and the adverse effects associated with immunosuppression. Cells can be seeded onto biodegradable polymer scaffolds with three-dimensional structures, which can subsequently be implanted into the body to support organ architecture and promote liver tissue regeneration [135].

Isolated hepatocytes often undergo cell death or loss of function due to the absence of cellular interactions and disruption of their native microenvironment. Collagen, therefore, provides hepatocytes with a microenvironment that closely resembles the native liver tissue, supporting liver tissue regeneration. One study utilized collagen bio-ink to establish an appropriate microenvironment for hepatocytes, HUVECs, and human lung fibroblasts. The bio-ink was injected into the ducts of polycaprolactone (PCL) frameworks, successfully creating a microenvironment that supported the co-cultivation of these cell types. This setup induced the formation of a capillary-like network, which enhanced hepatocyte protein secretion and metabolic activity [136]. Another study developed a bionic membrane composed of esterified collagen and chitosan methacrylate. Hepatocytes cultured on this collagen-integrated membrane exhibited a complete differentiation pattern, demonstrating superior growth, viability, and enhanced liver-specific functions relative to those cultured on chitosan methacrylate esterified-only membranes [137].

In addition to the simple encapsulation of cells within biomaterials, more advanced 3D models have been developed to better mimic liver physiology and generate intricate structures that replicate the architecture of liver tissue. These models can promote cell differentiation, proliferation, and migration, ultimately enhancing liver injury repair [138]. Gao et al. [139] utilized buckminsterfullerene tablets functionalized with collagen (Col-Nor), which were further loaded with cells to construct a centimeter-scale bioprinted liver micro-model. The gel’s diffusion and proliferation of HUVECs formed a vascularized network. In contrast, this network’s primary human hepatocyte (PHH) spheroids could produce liver-specific proteins and upregulate cytochrome activity, exhibiting functional liver tissue characteristics in vitro. In particular, demineralized collagen sponge scaffolds can be engineered to serve as scaffolds for in vitro liver models. Following the differentiation of rat small hepatocytes (SHs) into mature hepatocytes, an in vitro liver model can subsequently be reconstructed through interactions with liver nonparenchymal cells (NPCs) on collagen sponges [140]. HepG2 cells cultured on honeycomb-structured collagen sponges, which exhibit a significant increase in albumin mRNA expression levels, can be utilized for drug metabolism studies, toxicity testing, and screening of therapeutic agents [141]. Additionally, hydroxyapatite-mineralized collagen sponges can serve as carriers for the anti-hepatocellular carcinoma drug, the STAT-3 inhibitor WP1066, facilitating the sustained release of the drug in an in vitro hepatocyte model comprising HEPA1-6 and HepG2 cells. This system effectively inhibits the viability of hepatocellular carcinoma cells through the synergistic action of the drug and hydroxyapatite [142].

Moreover, in vitro liver models can be used to investigate cellular behavior under disease conditions or in response to changes in the microenvironment. A hepatic sinusoidal model was developed through coaxial extrusion 3D bioprinting, where the core compartment of the pre-vascular structure consisted of alginate and methylcellulose bio-ink loaded with HepG2 cells, which mimic the properties of human hepatocytes. The outer structure was printed using collagen/fibronectin bio-ink containing HUVECs and human dermal fibroblasts. The interactions between these different cell types in the culture model increased albumin secretion from HepG2 cells when co-cultured with HUVECs, while fibroblasts played a supportive role in angiogenesis [143].

### 5.6. Other Tissue Engineering

Collagen is the primary protein component in tissues such as tendons and ligaments. It has been extensively used in tendon tissue engineering to facilitate myogenic fiber regeneration and restore motor function [144]. Vascularization and innervation are critical factors for muscle regeneration and functional recovery. Incorporating cell-mediated microvascular networks into scaffolds represents a promising strategy to promote these processes. HUVECs were co-encapsulated with human mesenchymal stem cells within collagen hydrogels to treat muscle injuries. This approach not only accelerated muscle regeneration but also strengthened muscle innervation, promoted angiogenesis, and facilitated anastomosis, thereby contributing to the restoration of function in the injured muscle [145].

Cells and biomaterials are commonly used in vascular tissue engineering to construct near-native substitutes for cardiovascular tissue. Kim et al. [146] encapsulated fibroblasts in a collagen/alginate hydrogel for 3D bioprinting to replicate the structural characteristics of human blood vessels. The encapsulated cells exhibited a high survival rate of 92.13 ± 0.70%, but further in vivo studies are needed. Additionally, it has been shown that collagen scaffolds seeded with endothelial cells, when implanted into a murine carotid artery injury model, prevented in vivo cell inactivation, reduced injury-induced inflammation, and enhanced endothelialization and the reduction of smooth muscle proliferation [147]. Furthermore, constructing 3D tissue models of the microvascular system using collagen is valuable for preclinical drug screening and pathological studies. For instance, collagen/alginate scaffolds fabricated via an embedded printing method supported astrocyte branching and facilitated direct interaction with endothelial cells, a crucial aspect of neurovascular model development [148]. The arrangement and topology of collagen fibers within the hydrogel were also found to influence the organization of HUVECs in a microvascular model [149].

Biomaterials have been widely used in stomatology, particularly in periodontal regeneration, alveolar bone defect repair, and the healing of oral mucosal tissues. In these applications, collagen is pivotal in promoting rapid hemostasis, facilitating cell migration, and aiding bone regeneration [150,151]. Wang et al. [152] developed a double-layered scaffold composed of collagen and strontium-doped calcium silicate loaded with human gingival fibroblasts (hGFs). These collagen/strontium-doped calcium silicate bilayer scaffolds promoted the secretion of osteogenesis-related proteins by the human GFs. Upon implantation in animal models, the scaffolds supported the growth of new bone tissue, which progressively invaded the scaffold, ultimately facilitating periodontal regeneration. Additionally, human periodontal membrane fibroblasts (HPLFs) were loaded into collagen/riboflavin/dental light-emitting diode (LED) injectable hydrogels, resulting in the differentiation of the fibroblasts into myofibroblasts and pro-osteoblasts. This differentiation facilitated the targeted repair of periodontal tissues [153].

**Figure 3:**
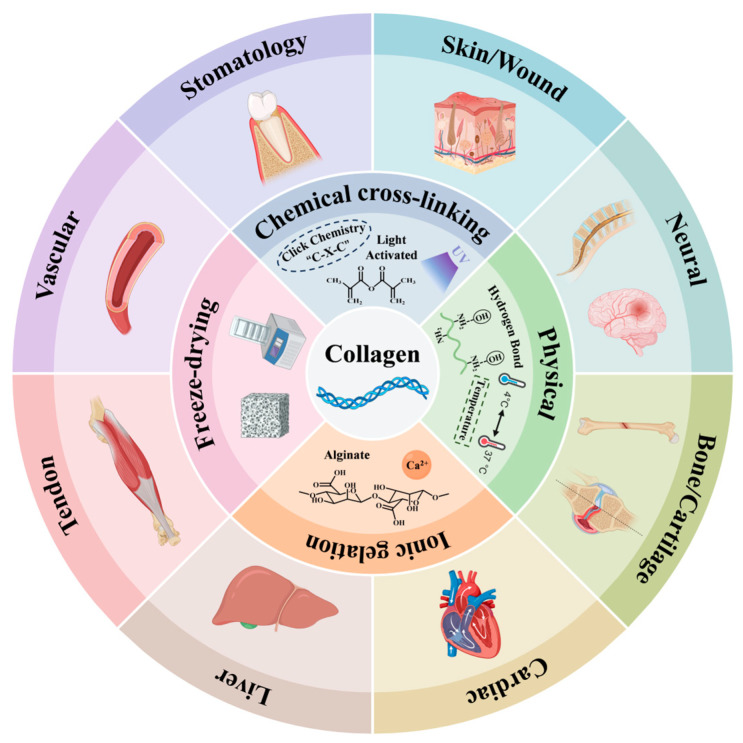
Schematic illustration of cell-loaded collagen scaffolds and their applications in various fields of tissue engineering.

Collagen scaffolds have been widely explored for regenerating various tissues, including the skin, bone, cartilage, nerve, heart, and liver (Figure 3). The microenvironment required for cell growth differs among these tissues. Collagen’s inherent properties as a natural extracellular matrix ensure its excellent biocompatibility, supporting effective tissue repair in various contexts. Table 3 summarizes the multiple applications of collagen scaffolds in tissue repair and regeneration, highlighting the key findings associated with their use.

## 6. Conclusions and Outlook

Collagen is one of the primary structural proteins in the vertebrate extracellular matrix, constituting approximately 25% of the total body protein. Its unique triple-helical structure, composed of collagen fibers, endows it with exceptional tensile strength and flexibility, providing essential structural support for cells and tissues. Collagen plays a crucial role in the coagulation pathway, facilitating platelet adhesion, promoting platelet aggregation, and facilitating thrombus formation, ultimately leading to hemostasis. Furthermore, it plays a pivotal role in wound healing, inflammation, and photoaging. Signal transduction via multiple molecular binding sites with cell surface integrins or DDRs synergistically influences its mechanical strength and fiber alignment, promoting cell adhesion, migration, proliferation, and differentiation.

Collagen scaffolds can be cross-linked through chemical methods, such as click chemistry, methacrylation reactions, or enzyme-mediated cross-linking; through physical methods, including intermolecular hydrogen bonding or temperature-induced phase transitions; or through ion-actuated gelation or freeze-drying into a porous sponge. These processes enhance the structural strength of the scaffolds, regulate their degradation rate, maintain cellular activity, improve stability, and reduce the likelihood of scaffold shrinkage or collapse. In tissue engineering, cells are loaded into collagen scaffolds and subsequently implanted into organisms, effectively integrating cell therapy and tissue engineering for tissue repair. These scaffolds are widely used in repairing skin, nerves, bones, cartilage, hearts, livers, and other tissues. Numerous studies have extended the functionality of collagen scaffolds by biofunctionalizing them with antimicrobial and antioxidant materials and incorporating active ingredients such as growth factors, extracellular vesicles, exosomes, and drugs, thereby conferring multifunctionality to the scaffolds and improving their reparative efficacy [154,155]. However, collagen scaffolds encounter challenges in clinical applications, requiring further advancements to enable effective translational use. Although collagen and its degradation products, such as amino acids, are non-toxic and biodegradable, the cellular sources and additional materials incorporated to enhance the performance of collagen scaffolds may provoke adverse biological reactions. This highlights the importance of carefully considering design to minimize host responses.

In conclusion, collagen tissue engineering applications represent a promising therapeutic approach for repairing tissue and organ damage, addressing challenges such as the organism’s limited self-healing capacity and the limitations of organ transplantation while offering innovative opportunities to integrate cellular therapies. However, the technology of implanting cell-laden scaffolds into the human body is still in its developmental stages and requires further research and optimization. Overcoming these challenges is crucial to advancing the clinical translation of collagen scaffolds and unlocking their full potential in tissue engineering and regenerative medicine.

## Figures and Tables

**Table 1:** The multiple cells and preparation methods for collagen scaffold encapsulation.

Method	Scaffold	Cross-Linking Agent or Influencing Factor	Encapsulated Cells	Reference
Click Chemistry	Thiolated-collagen injectable hydrogel	Thiol-alkene click chemistry	BMSCs	[61]
	Thiolated-collagen injectable hydrogel	Thiol-alkene click chemistry	BMSCs, HUVEC	[62]
	Hyaluronic acid/collagen hydrogel	SPAAC	Corneal epithelial cells	[64]
	EGF/collagen scaffolds	SPAAC	Corneal epithelial cells	[65]
Photoactivated Cross-linking	CSMA-COLMA hydrogel	Methacrylic anhydride	Keratinocytes, Fibroblasts	[67]
	Col-II-MA hydrogel	Methacrylic anhydride	BMSCs	[68]
	Collagen scaffolds	Riboflavin	Fibroblasts	[70]
	Collagen/hyaluronic acid scaffolds	Riboflavin	BMSCs	[71]
Enzyme Cross-linking	Collagen-hyaluronic acid hydrogel	HRP	HMEC, Fibroblasts	[73]
	Collagen fibril hydrogel	MTG	Fibroblasts	[77]
	Transglutaminase-cross-linked collagen hydrogels (Col-Tgel)	MTG	HDPSCs	[78]
Physical Force	Rapid sol-gel reversible thermosensitive collagen (RRTC) hydrogel	pH/Temperature	L929, HUVEC, Mouse myeloma cells (Sp2/0), MOVAS, IEC-6, HC	[81]
	Collagen hydrogel	pH/Temperature	MSC	[56]
	3D biomimetic collagen scaffolds	pH/Temperature	NHDFs, C2C12	[82]
Ionic Interaction Gelation	Alginate-collagen interpenetrating network (IPN) hydrogel	CaCl_2_	IDG-SW3	[86]
	Collagen-alginate microgels	CaCl_2_	HS-5, MDA-MB-231	[87]
	Sodium alginate/collagen hydrogel	CaCl_2_	hUC-MSCs	[88]
	Collagen-alginate 3D microscaffolds	CaCl_2_	MG-63, MDA-MB-231, U2OS, HCT-116	[89]
Freeze-drying	Cellulose and collagen nano-scaffold	Freeze-drying	BMSCs and differentiated keratinocytes	[94]
	Double-layer collagen sponge	Freeze-drying	C2C12/MC3T3-E1	[95]

**Table 2:** Advantages and disadvantages of cell-loaded collagen scaffold preparation methods.

Method	Advantages	Disadvantages	Reference
Click Chemistry	Highly efficient Highly specific covalent bonding Non-cytotoxic properties It is suitable for in situ cross-linking in living tissue	Requires that natural polymers incorporate click groups	[60,61,62,65]
Photoactivated Cross-linking	Mild reaction conditions In situ gelation The modulation of mechanical properties is facilitated Applicable to 3D bioprinting of live cells	Restricted light penetration depth	[66,69,70,71]
Enzyme Cross-linking	Absence of exogenous reagent residues Biocompatibility Preserves the triple helix structure Elevated specificity	Reaction rate limitation: LOX Inadequate mechanical properties	[73,74,75,77]
Physical Force	Absence of chemical modification Rapid and reversible molding Facile handling	Inadequate mechanical properties Necessitates precise temperature regulation	[56,79,81]
Ionic Interaction Gelation	Rapid prototyping Synergistic improvement in mechanical properties Increases cell adhesion sites	The pH sensitivity of collagen must be tuned to a neutral equilibrium for effective cross-linking	[83,84,85,86,89]
Freeze-drying	Tunable pore size Absence of exogenous chemical residues Straightforward preparation process Swift absorption of wound exudate	Inadequate mechanical properties Accelerated degradation rates Long-term stability and storage problems	[90,91,92,93,94]

**Table 3:** Cell-loaded collagen scaffolds for tissue engineering applications.

Tissue/Organ to Be Regenerated	Scaffold	Encapsulated Cells	Key Findings	Reference
Skin/Wound Healing	Bilayered PCL/gelatin nanofibers-alginate/collagen hydrogel	ADSCs	The bilayer scaffold containing ADSCs reduced inflammation and improved re-epithelialization and collagen reorganization.	[96]
	A novel cellulose and collagen nano-scaffold	Bone marrow-derived mesenchymal stem cells and differentiated keratinocyte-like cells	Cell therapy did not induce inflammation; combining scaffold and cellular therapy enhanced collagen deposition.	[94]
	Thermosensitive injectable chitosan/collagen/β-glycerophosphate composite hydrogels	MSCs	The combined treatment accelerated the wound closure in diabetic mice by enhanced vascularization and paracrine effects.	[97]
	Collagen dermal-replacement scaffolds	BMSCs	CBS-MSCs facilitated the noncontractile and re-epithelialization processes, as well as granulation tissue regeneration and neovascularization, in chronic diabetic wounds.	[98]
	A collagen–glycosaminoglycan matrix to form a dermis-like tissue sheet	MSCs	Prolonged MSC engraftment is associated with accelerated wound re-epithelialization and healing, accompanied by increased macrophage recruitment and angiogenesis.	[99]
Nerve	Collagen-fibrin hydrogel	mNSC	Enhanced cognitive function through the reconstruction of the damaged cortex.	[103]
	Collagen/heparan sulfate porous scaffolds	NSCs	Enhanced regeneration of neurons, nerve fibers, synapses, and myelin sheaths in injured brain tissue, reduced brain edema and apoptosis, and substantially recovered motor and cognitive functions.	[104]
	Methacrylate hyaluronic acid/collagen hydrogel	MSCs	Maintained disc height, protected NP, and alleviated vascularization and inflammation.	[106]
	Type I collagen hydrogel	MSCs	It effectively prevents disc degeneration and inhibits cell apoptosis following discectomy.	[107]
	Collagen sponge scaffold	hbNSPCs hscNSPCs	Effectively promoted long-term cell survival and neuronal differentiation and improved the SCI microenvironment by reducing inflammation and glial scar formation.	[109]
	Collagen/silk fibroin scaffold	NSCs	Significantly increased the amplitude of motor-evoked potentials, as well as improved continuity and cavity filling within the injured spinal cord.	[110]
Bone/Cartilage	HAP/collagen scaffold	BMSCs	BMSCs on the scaffold remained viable and continued to proliferate, exhibiting high alkaline phosphatase expression.	[117]
	Collagen-HAP scaffold	hADSCs	Capable of recruiting host cells to undergo osteogenic differentiation while promoting bone enlargement and the formation of vascular elements.	[118]
	Collagen/strontium-doped bioactive glasses/HAP nanorods scaffold	OBs	Positively influenced cell proliferation and metabolic activity, enhancing alkaline phosphatase activity in osteoblasts and reducing osteoclast differentiation.	[119]
	Collagen/PVA-CaCO₃ membranes	BMSCs	Effectively promoted BMSC adhesion, proliferation, and osteogenic differentiation.	[121]
	Collagen/PLGA/HAP fibrous scaffolds	MSCs	Collagen coating enhanced cell–membrane interactions.	[122]
	Collagen/phosphorous-modified polycaprolactone porous scaffold	AD-MSCs	Facilitates cell adhesion, proliferation, and upregulation of osteogenic marker genes, thereby inducing osteogenic differentiation of stem cells.	[123]
	Unidirectional collagen-dECM scaffolds	BMSCs	Highlights the immature cartilage and dECM at different developmental stages, which result in the diversified effects of BMSCs.	[124]
	Collagen/silk fibroin scaffold	ADSCs	Stimulates chondrogenic differentiation of stem cells and enhances cartilage regeneration.	[125]
Cardiac	Collagen-PEDOT–PSS hydrogel	Human-induced pluripotent stem cell (hiPSC)-derived cardiomyocytes	Facilitates partial regeneration of cardiac muscle, improving contractility, calcium handling, and conduction efficiency.	[129]
	Collagen-hyaluronic acid hydrogels	Human embryonic stem cell-derived cardiomyocytes	Exhibits heat sensitivity, self-repair capability, and electrical conductivity, facilitating myocardial regeneration.	[130]
	Gelatin-methacryloyl–collagen hydrogel	hCM, hCF	Vessel formation and stabilization, reduced fibrosis, increased left ventricle thickness, and enhanced cardiac function.	[131]
	Collagen bio-ink	Human cardiomyocytes	The printed heart model demonstrates synchronized contraction, directed action potential propagation, and wall thickening during peak ventricular contraction.	[133]
Liver	Collagen hydrogel	HCs, HUVEC, Human lung fibroblasts	Constructs liver tissue structures that facilitate interaction between heterogeneous cells.	[136]
	Methacrylated chitosan/jellyfish collagen membranes	Hepatocytes	Provides an optimal microenvironment for liver cells.	[137]
	Norbornene-functionalized collagen (Col-Nor) hydrogel	HUVEC	Development of a three-dimensional (3D) liver tissue model incorporating branching vascular networks.	[139]
	Demineralized collagen sponge	SHs, NPCs	Hepatic organoids can be rapidly reconstructed in a collagen sponge by rat SHs and NPCs.	[140]
	Demineralized collagen sponge	HepG2	The collagen sponge can be utilized for drug metabolism studies, toxicity testing, and therapeutic agent screening.	[141]
	HAP-mineralized collagen sponges	HEPA1-6, HepG2	Collagen matrix scaffold as a vehicle in an in vitro hepatocellular model.	[142]
	Alginate/methylcellulose bio-ink–collagen/fibronectin bio-ink	HepG2, HUVEC, Human dermal fibroblasts	Development of an In vitro Triple-Culture Model with Complex Hepatic Sinusoids.	[143]
Tendon	Collagen hydrogel	HUVEC, MSCs	Development of large vascularized neural tissue structures for repairing volumetric muscle loss and restoring muscle function.	[145]
Vascular	Tyramine-functionalized alginate-collagen hybrid hydrogel	Fibroblast cells	Three-dimensional (3D) printed vascular extracellular matrix (ECM) mimetic scaffolds for supporting tissue regeneration.	[146]
	Collagen scaffold	ECs	It prevents in vivo cell inactivation, reduces injury-induced inflammation, and enhances endothelialization and smooth muscle proliferation.	[147]
	Alginate/collagen hydrogels	Mouse brain microvascular endothelial cells (BEND.3), Astrocytes	A 3D hollow coaxial neurovascular model is fabricated.	[148]
	Collagen hydrogels	HUVEC	Developing organ-on-a-chip and 3D tissue models with complex microvasculature.	[149]
Stomatology	Collagen/strontium-doped calcium silicate scaffold	hGF	Guided periodontal regeneration.	[152]
	Collagen/riboflavin hydrogels	HPLFs	Well-oriented periodontal ligament and alveolar bone regeneration.	[153]

## Abbreviations

The following abbreviations are used in this manuscript:

Abbreviation	Full Name
MMPs	Matrix metalloproteinases
TIMPs	Tissue inhibitors of metalloproteinases
DDRs	Discoidin Domain Receptors
SPAAC	Strain-promoted azide-alkyne cycloaddition
COLMA	Collagen methacrylate
CSMA	Chondroitin sulfate methacrylate
Col-II-MA	Type II collagen methacrylamide
RF	Riboflavin
LOX	Lysine oxidase
MTG	Glutamine transaminase
HRP	Horseradish peroxidase
LCST	Low Critical Solution Temperature
UCST	Upper Critical Solution Temperature
SBF	Simulated body fluid
IPNs	Interpenetrating Polymer Networks
ESCs	Embryonic stem cells
BMSCs	Bone marrow mesenchymal stem cells
HUVECs	Human umbilical vein endothelial cells
HDPSCs	Human dental pulp stem cells
L929	Mouse fibroblasts
MOVASs	Murine aortic vascular smooth muscle cells
IEC-6	Rat intestinal epithelial cells
HCs	Human chondrocytes
MSC	Mesenchymal stem cell
NHDFs	Normal Human Dermal Fibroblasts
C2C12	Murine myoblasts
IDG-SW3	Murine bone cells
HS-5	Human fibroblast cell line
MDA-MB-231	Epithelial adenocarcinoma cells
hUC-MSCs	Human umbilical cord mesenchymal stem cells
MG-63	Human osteosarcoma cells
U2OS	Human osteosarcoma clonal cells
HCT-116	Human colon cancer cells
NSCs	Neural stem cells
PCL/Gel	Polycaprolactone/Gelatin
Col/Alg	Collagen/Alginate
ADSCs	Adipose-derived Stem Cells
CDRSs	Collagen dermal replacement scaffolds
EDS	Engineered dermal substitute
CNS	Central Nervous System
TBI	Traumatic brain injury
SCI	Spinal cord injury
mNSC	mouse Neural stem cell
NP	Nucleus pulposus
AF	Annulus fibrosus
IDD	Intervertebral Disc Degeneration
hbNSPCs	Human brain-derived neural stem/progenitor cells
hscNSPCs	Human spinal cord-derived neural stem/progenitor cells
ACSS	Aligned collagen sponge scaffold
HAP	Hydroxyapatite
hADSCs	Human adipose-derived stem cells
P-PCL	Phosphorus-modified polycaprolactone
AD-MSCs	Adipose-derived mesenchymal stem cells
ALP	Alkaline phosphatase
OBs	Osteoblasts
OCs	Osteoclast precursors
dECM	Decellularized extracellular matrix
ECM	Extracellular matrix
MI	Myocardial infarction
hiPSC	Human-induced pluripotent stem cell
APTES	3-Aminopropyl-triethoxysilane
hCMs	Human ventricular cardiomyocytes
hCFs	Human cardiac fibroblasts
AMI	Acute myocardial infarction
FRESH	Freeform reversible embedding of suspended hydrogels
PCL	Polycaprolactone
PHHs	Primary human hepatocytes
HCs	Hepatocytes
ECs	Endothelial cells
hGFs	Human gingiva fibroblasts
HPLFs	Human periodontal membrane fibroblasts
